# Supplementary material for: Molecular Insights into Outer Dynein Arm Defects in Primary Ciliary Dyskinesia: Involvement of ZMYND10 and GRP78
Source: Cells. 2025 Jun 17;14(12):916. doi: 10.3390/cells14120916 (PMC12190660; doi:10.3390/cells14120916)
Supplement: Supplementary file 1 [file cells-14-00916-s001.zip › Supplementary Tables.pdf]

Supplementary Table S1. List of IF antibodies

	Source	Company	Dilution
**Primary Antibodies**			
DNAH5	Rabbit	Sigma (HPA037470)	1:200
β tubulin	Mouse	Sigma (T8328)	1:200
β tubulin	Rabbit	ABclonal (AC008)	1:200
ZMYND10	Mouse	Santa Cruz (sc398350)	1:200
GRP78	Mouse	abcam (ab212054)	1:200
**Secondary Antibodies**			
Alexa fluor 594	Goat anti-rabbit	Thermo (A32740)	1:1000
Alexa fluor 488	Goat anti-mouse	abcam (ab150113)	1:1000

Supplementary Table S2: Genetic Characteristics of the Individuals

**İndividual ID**	**Sex**	**Age**	**Gen**	**Mutation: Coding (HGVS nomenclature c.)**	**Mutation: Protein (HGVS nomenclature p.)**	**Type**	**Zygosity**
1	M	21y	*DNAH5*	c.11740G>A	p.Glu3914Lys	Missense	Comp. Het.
2	F	17y	*DNAH5*	c.13486C>T	p.Arg4496Ter	Nonsense	Hom
3	F	22 y	*DNAH5*	c.7615T>C	p.Trp2539Arg	Missense	Hom
4	F	18y	*DNAH5*	c.8897C>T	p.Thr2966Met	Missense	Hom
5	F	19y	*DNAH5*	c.5747G>A	p.W1916*	Nonsense	Het
6	M	16y	*DNAH5*	c.2710G>T	p.Glu904Ter	Nonsense	Hom
7	F	16y	*DNAH5*	c.2368G>C	p.Ala790Pro	Missense	Hom
8	M	14y	*DNAH5*	c.9502C>T	p.R3168*	Nonsense	Hom
